# Attack-Aware Secure Sensor Transmission Using Pinching- Antenna Systems and Trust-Guided Cooperative Jamming

**DOI:** 10.3390/s26175331

**Published:** 2026-08-22

**Authors:** Shen Qian, Zhao Li, Meng Cheng

**Affiliations:** 1 Department of Information Systems, Faculty of Informatics, Tokyo City University, Yokohama 224-8551, Japan; 2 School of Cyber Engineering, Xidian University, Xi’an 710126, China; 3 The College of Information, Mechanical and Electrical Engineering, Shanghai Normal University, Shanghai 201418, China

**Keywords:** intelligent sensor networks, attack detection, compromised relay sensor, physical-layer security, pinching-antenna systems, cooperative jamming, trust-aware communication, secure sensing-data transmission

## Abstract

Intelligent sensor networks are increasingly deployed in security-critical environments, where confidential sensing data may be forwarded through low-security relay sensors that are vulnerable to compromise, misuse, or internal eavesdropping. This paper investigates attack-aware secure sensor transmission in a sensor network enabled by a pinching-antenna system (PASS) with a potentially compromised relay sensor. The relay sensor assists data forwarding from a source sensor to a destination fusion center, but its received signal may also cause internal information leakage. To mitigate this threat, a trust-aware cooperative jamming framework is proposed, in which a cooperative security node generates artificial noise, feeds it into the PASS waveguide, and radiates it through selected pinching antennas toward the untrusted relay while limiting interference leakage to the legitimate fusion center. A trust-aware cooperation coefficient is introduced to characterize the security-response capability of the cooperative security node, and a candidate-set-aided PASS activation and jamming-power allocation algorithm is developed for secure sensing-data delivery. Reliable-and-secure probability, secrecy outage probability, and attack-aware secure delivery probability are adopted to jointly evaluate data reliability, confidentiality, and relay-attack resilience. Numerical results show that the proposed scheme improves secure transmission performance compared with fixed-antenna jamming, random PASS activation, trust-unaware PASS, and no-jamming benchmarks. The results also indicate that a detection-triggered PASS response can maintain a higher secure delivery probability as the relay attack probability increases.

## 1. Introduction

Intelligent sensor networks and Internet of Things (IoT) systems have become important infrastructures for industrial monitoring, smart cities, healthcare, environmental sensing, transportation, and emergency-response applications [1,2,3,4]. In these scenarios, sensor nodes often have limited transmit power, constrained computation capability, and unstable direct-link quality. Therefore, confidential sensing data may need to be forwarded through intermediate relay sensors before reaching a destination fusion center. However, relay-assisted sensing also enlarges the attack surface of the network. Sensor and IoT systems are known to face security, privacy, trust, routing, denial-of-service, intrusion, anomaly, and compromised-node threats [5,6,7,8,9,10,11,12,13,14,15,16]. A relay sensor may be compromised, misused, or behave as an honest-but-curious node that continues forwarding data while attempting to infer confidential sensing information. Therefore, secure sensor data transmission requires not only reliable data delivery but also protection against internal information leakage and attack-triggered abnormal behavior.

Physical-layer security provides a complementary protection mechanism by exploiting the randomness, spatial selectivity, and controllability of wireless channels [17,18,19,20,21,22]. In contrast to purely upper-layer cryptographic approaches, physical-layer security aims to improve confidentiality by utilizing propagation characteristics, artificial noise, cooperative transmission, and channel-aware resource allocation. In untrusted relay networks, the relay may be helpful for improving communication reliability but harmful from the secrecy perspective because its received signal can become a source of information leakage. Cooperative jamming and artificial noise have therefore been studied as effective approaches for degrading the reception quality of untrusted relays or eavesdroppers [23,24,25,26,27]. However, most existing cooperative-jamming studies assume fixed antenna deployments and fully cooperative helpers, which may be restrictive for distributed sensor networks with heterogeneous trust levels, limited energy budgets, and attack-dependent security requirements.

Reconfigurable wireless environments have recently attracted substantial attention because they can reshape propagation channels by controlling passive or semi-passive electromagnetic structures [28,29,30,31,32,33,34]. Intelligent reflecting surfaces and related reconfigurable structures have also been investigated for physical-layer security by strengthening legitimate links or suppressing information leakage toward eavesdroppers [35,36]. More recently, pinching-antenna systems (PASS) have emerged as a flexible antenna architecture for wireless channel reconfiguration [37,38,39,40]. A PASS employs a dielectric waveguide as the signal transmission medium and forms reconfigurable radiating points by activating pinching antennas along the waveguide. Compared with conventional fixed-antenna systems, PASS provides a new spatial degree of freedom because the effective wireless channel can be shaped by selecting different pinching-antenna positions. This feature is particularly attractive for secure sensor networks, where artificial noise should be directed toward a suspicious relay sensor while its interference leakage to the destination fusion center should be controlled. Although recent studies have investigated PASS from the perspectives of channel modeling, beamforming, channel estimation, and capacity characterization, its application to attack-aware secure sensor transmission remains insufficiently explored.

Recent studies in sensor and IoT security have also investigated physical-layer security, interference mitigation, jamming detection, and secure multi-hop transmission in wireless sensor and IoT environments [41,42,43]. These works are closely related to secure wireless sensing and communication, whereas the present paper focuses on a PASS-assisted physical-layer security response after a trust or attack-risk indication has been obtained. In contrast, physical-layer security studies on untrusted relaying and cooperative jamming mainly focus on secure wireless transmission under information leakage, but they usually do not connect the wireless security response with trust or attack-risk indications in intelligent sensor networks. Meanwhile, reconfigurable wireless environments and PASS provide new spatial degrees of freedom for channel shaping, but their use for attack-aware secure sensor transmission with a potentially compromised relay sensor remains underexplored.

To address this gap, this paper investigates attack-aware secure sensor transmission in a PASS-enabled intelligent sensor network with a potentially compromised relay sensor. The relay sensor assists data forwarding from a source sensor to a destination fusion center, but its first-phase received signal is treated as a potential source of information leakage. A cooperative security node equipped with PASS generates artificial noise, feeds it into the dielectric waveguide, and radiates it through selected pinching antennas to suppress relay-side information leakage while controlling interference leakage to the destination. To connect the physical-layer security response with intelligent sensor security, a trust-aware cooperation coefficient is introduced to represent the willingness or capability of the cooperative security node to participate in secure cooperative jamming. In addition, an attack-aware secure delivery metric is considered to evaluate the effect of relay attack probability and detection-triggered security response.

The main contributions are summarized as follows:

- A PASS-enabled secure sensor transmission model is established for a relay-assisted sensing scenario, where the relay sensor contributes to data forwarding but is also treated as a possible internal information-leakage point.
- A cooperation-aware jamming model is developed by linking the effective artificial-noise power to a security-response coefficient. This model captures different trust, energy, and attack-risk conditions without assuming fully altruistic cooperation by the cooperative security node.
- A candidate-set-aided PASS activation algorithm is proposed. The algorithm combines metric-driven, relay-oriented, destination-protection, random, and warm-start candidates, and then selects the active pinching-antenna set and jamming power according to either reliable-and-secure probability (RSP) maximization or secrecy outage probability (SOP) minimization.
- In addition to RSP and SOP, an attack-aware secure delivery probability is introduced to evaluate how detection-triggered low-response and high-response modes affect secure delivery under different relay attack probabilities.
- Simulation results clarify the impact of the cooperation coefficient, jamming-power budget, number of activated pinching antennas, and relay attack probability on secure sensing-data transmission.

The remainder of this paper is organized as follows. Section 2 presents the system model, including the network and security model, trust-aware relay attack model, PASS-assisted channel model, and two-phase transmission protocol. Section 3 introduces the trust-aware secure sensor transmission framework and defines the equivalent transmission rates, reliable-and-secure probability, secrecy outage probability, and attack-aware secure delivery probability. Section 4 formulates the empirical RSP- and SOP-oriented optimization problems and presents the proposed candidate-set-aided PASS activation and jamming-power allocation algorithm. Section 5 provides numerical results to evaluate the effects of the trust-aware cooperation coefficient, maximum jamming power, number of activated pinching antennas, and relay attack probability. Section 6 discusses the limitations and practical considerations of the proposed framework. Finally, Section 7 concludes the paper and outlines future research directions.

## 2. System Model

### 2.1. Network and Security Model

**Figure 1:**
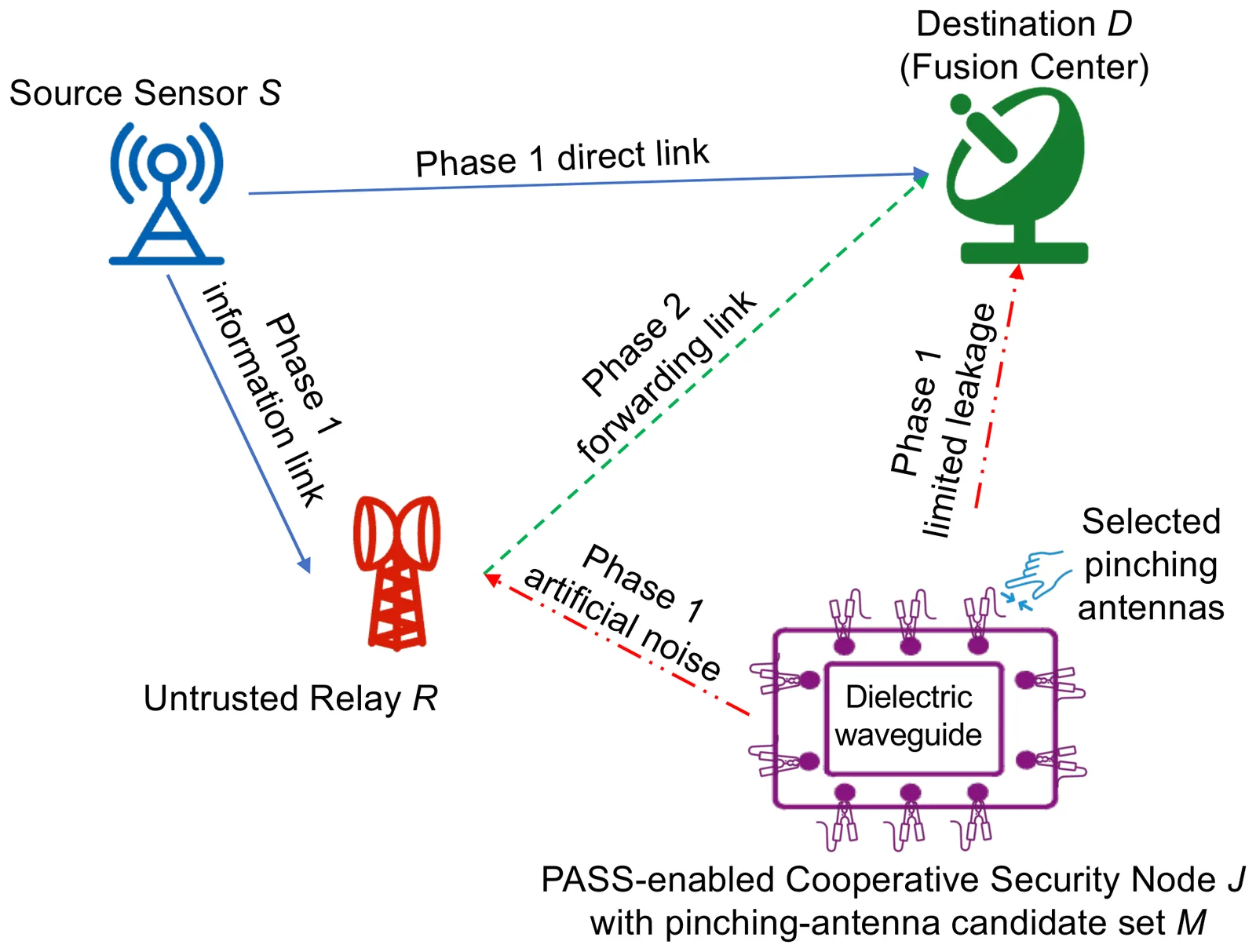
System model of the PASS-enabled intelligent sensor network with an untrusted relay sensor. In Phase 1, the source sensor *S* transmits confidential sensing data to the relay sensor *R* and also provides a direct link to the destination fusion center *D*. The PASS-enabled cooperative security node *J* generates artificial noise, feeds it into the dielectric waveguide, and radiates it through the selected pinching antennas to suppress information leakage at the potentially compromised relay sensor. A limited amount of artificial-noise leakage may also reach the fusion center. In Phase 2, the relay sensor forwards a re-encoded signal to the fusion center, while node *J* remains silent.

We consider a PASS-enabled intelligent sensor network consisting of a source sensor *S*, an untrusted relay sensor *R*, a destination fusion center *D*, and a cooperative security node *J* equipped with a pinching-antenna system (PASS), as shown in Figure 1. The security node acts as a friendly jammer by generating artificial noise, feeding it into the PASS waveguide, and radiating it through selected pinching antennas when a secure transmission response is required. The source sensor *S* collects confidential sensing data and broadcasts the confidential signal in Phase 1, which can be observed by both the relay sensor *R* and the destination fusion center *D* through the relay and direct links, respectively. The relay sensor *R* assists the data delivery due to the limited direct transmission capability of *S*, but it may be compromised by an internal attacker or behave as an honest-but-curious relay that attempts to infer confidential sensing information from its received signal.

The PASS-enabled cooperative security node *J* provides a physical-layer security response through selected pinching antennas distributed along a dielectric waveguide. Different from a fixed-antenna jammer, PASS enables flexible activation of radiating points, thereby shaping the artificial-noise channel toward the untrusted relay sensor while limiting interference leakage to the fusion center.

The considered security model includes the following components:

- Internal relay attack: The relay sensor *R* is potentially compromised and may exploit its received signal to infer confidential sensing data.
- Trust-aware security response: The cooperative security node *J* adjusts its jamming power and PASS activation according to the trust or cooperation level associated with the legitimate sensor pair.
- PASS-assisted adaptive jamming: A subset of candidate pinching antennas is activated to radiate artificial noise in Phase 1, thereby improving the secrecy performance of sensor data transmission.

For tractability, the continuous PASS waveguide is discretized into *M* candidate pinching-antenna positions. Let

(1) M={1,2,…,M}

denote the set of candidate positions. The binary variable $a_{m}\in \left\{0,1\right\}$ indicates whether the *m*-th pinching antenna is activated. The active set is denoted by

(2) $$A=\{m\in M|a_{m}=1\}.$$

When PASS-assisted jamming is enabled, at least one pinching antenna is activated. The PASS activation constraint is given by

(3) $$1\leq \sum_{m=1}^{M}a_{m}\leq K_{\max},$$

where $K_{\max}$ is the maximum number of activated pinching antennas.

### 2.2. Trust-Aware Relay Attack Model

To connect the physical-layer security design with attack-aware intelligent sensing, we introduce a trust-aware relay attack indicator. Let $\theta_{R}\in \left\{0,1\right\}$ denote the relay security state, where $\theta_{R}=0$ represents a normal relay sensor and $\theta_{R}=1$ represents a compromised or suspicious relay sensor.

The cooperative security node *J* is assumed to have access to a trust score of the relay sensor, which may be obtained from local observations, historical forwarding behavior, physical-layer measurements, or an external attack-detection interface. The trust score is denoted by

(4) $$T_{R}\in \left[0,1\right],$$

where a larger $T_{R}$ indicates a more trustworthy relay sensor. The corresponding attack-risk level is defined as

(5) $$\varphi_{R}=1-T_{R}.$$

In a simple risk-triggered implementation, the cooperation coefficient can be mapped from the attack-risk level as

(6) $$\tau =\tau_{\min}+\left(\tau_{\max}-\tau_{\min}\right)\varphi_{R},$$

where $0\leq \tau_{\min}\leq \tau_{\max}\leq 1$. This expression indicates that a higher estimated relay-attack risk triggers a stronger cooperative jamming response. In the numerical evaluation, τ is varied as a system parameter to study the impact of different trust-aware response levels.

In this work, the detailed trust-estimation algorithm is not the main optimization target; instead, we focus on the physical-layer security response after a trust or attack-risk level has been obtained. Unless otherwise specified, τ is treated as a system-level trust-aware cooperation parameter and is later incorporated into the effective jamming power. A larger τ indicates a stronger willingness or capability of the cooperative security node to participate in secure cooperative jamming.

### 2.3. Channel Model

The direct wireless channel between node *i* and node *j* is denoted by $h_{i,j}$. For the PASS-assisted jamming link, the equivalent baseband channel from the selected pinching antennas of node *J* to node u∈{R,D} is modeled as

(7) $$g_{u}\left(a\right)=\chi \left(a\right)\sum_{m=1}^{M}a_{m}\rho_{m}{\tilde{h}}_{m,u},$$

where $\rho_{m}$ represents the propagation response from the feed of node *J* along the dielectric waveguide to the *m*-th pinching antenna, and ${\tilde{h}}_{m,u}$ denotes the wireless channel from the *m*-th pinching antenna to node *u*. Thus, node *J* generates and feeds the artificial-noise signal, while the selected pinching antennas act as the actual wireless radiating points.

The normalization factor χ(a) is used to ensure that PASS activation changes the spatial jamming channel but does not change the total effective jamming power. Unless otherwise specified, it is given by

(8) $$\chi \left(a\right)={\left(\sum_{m=1}^{M}a_{m}{\left|\rho_{m}\right|}^{2}\right)}^{-\frac{1}{2}}.$$

The waveguide propagation response is expressed as

(9) $$\rho_{m}=\sqrt{\eta_{m}}e^{-\kappa l_{m}}e^{-j\frac{2\pi }{\lambda_{g}}l_{m}},$$

where $\eta_{m}$ is the radiation efficiency of the *m*-th pinching antenna, κ is the amplitude attenuation coefficient along the waveguide, $l_{m}$ is the propagation distance from the feed point to the *m*-th pinching antenna, and $\lambda_{g}$ is the guided wavelength.

The factor $e^{-\kappa l_{m}}$ accounts for the amplitude loss accumulated from the waveguide feed to the *m*-th pinching antenna; accordingly, κ is defined here as an amplitude attenuation coefficient. Because $\eta_{m}$ is a power-efficiency ratio, its square root appears in the complex baseband amplitude coefficient, so that $|\sqrt{\eta_{m}}{|}^{2}=\eta_{m}$.

The wireless channel from the *m*-th pinching antenna to node *u* is modeled as

(10) $${\tilde{h}}_{m,u}=\sqrt{\beta_{0}{\left(d_{m,u}/d_{0}\right)}^{-\alpha_{u}}}e^{-j\frac{2\pi }{\lambda }d_{m,u}}\xi_{m,u},$$

where $\beta_{0}={\left(\lambda /\left(4\pi d_{0}\right)\right)}^{2}$ is the dimensionless free-space channel power gain at the reference distance $d_{0}=1$ m, $d_{m,u}$ is the distance between the *m*-th pinching antenna and node *u*, $\alpha_{u}$ is the path-loss exponent, λ is the carrier wavelength, and $\xi_{m,u}$ denotes the small-scale fading coefficient. For a line-of-sight dominated PASS link, $\xi_{m,u}=1$ can be used as a simplified deterministic model.

### 2.4. Transmission Protocol

The secure sensor data transmission is divided into two half-duplex phases.

Phase 1: Sensing-data transmission and cooperative jamming. The source sensor *S* transmits the confidential sensing signal $x_{S}$, while the cooperative security node *J* generates the artificial-noise signal $x_{J}$ and feeds it into the PASS. The selected pinching antennas then radiate the artificial noise in Phase 1 with effective power $P_{J}^{\mathrm{eff}}$ to degrade information leakage at the untrusted relay sensor. The radiated artificial noise primarily targets the relay sensor, while a limited amount of leakage may also reach the fusion center. The trust-aware model of $P_{J}^{\mathrm{eff}}$ will be specified in Section 3. The received signals at *R* and *D* are respectively given by

(11) $$y_{R}^{\left(1\right)}=\sqrt{P_{S}}h_{S,R}x_{S}+\sqrt{P_{J}^{\mathrm{eff}}}g_{R}\left(a\right)x_{J}+n_{R}^{\left(1\right)},$$

and

(12) $$y_{D}^{\left(1\right)}=\sqrt{P_{S}}h_{S,D}x_{S}+\sqrt{P_{J}^{\mathrm{eff}}}g_{D}\left(a\right)x_{J}+n_{D}^{\left(1\right)},$$

where $P_{S}$ is the transmit power of the source sensor, $P_{J}^{\mathrm{eff}}$ is the effective artificial-noise power radiated through the selected pinching antennas, $x_{S}$ and $x_{J}$ are normalized as $E[|x_{S}{|}^{2}]=E[|x_{J}{|}^{2}]=1$, and $n_{R}^{\left(1\right)}∼\mathrm{CN}\left(0,\sigma_{R}^{2}\right)$ and $n_{D}^{\left(1\right)}∼\mathrm{CN}\left(0,\sigma_{D}^{2}\right)$ are additive white Gaussian noises.

Phase 2: Relay forwarding. The relay sensor *R* forwards a re-encoded signal $x_{R}=f\left(y_{R}^{\left(1\right)}\right)$ to the fusion center *D*, where f(·) denotes the lossy or partial forwarding operation and $x_{R}$ is normalized as $E[|x_{R}{|}^{2}]=1$. The relay is assumed to maintain forwarding functionality, but its first-phase observation may still be exploited for internal information leakage. The cooperative security node remains silent in this phase, and no artificial noise is radiated by the selected pinching antennas, so as to avoid degrading the relay-to-destination forwarding link. The received signal at *D* is given by

(13) $$y_{D}^{\left(2\right)}=\sqrt{P_{R}}h_{R,D}x_{R}+n_{D}^{\left(2\right)},$$

where $P_{R}$ is the relay transmit power and $n_{D}^{\left(2\right)}∼\mathrm{CN}\left(0,\sigma_{D}^{2}\right)$.

## 3. Trust-Aware Secure Sensor Transmission

### 3.1. Trust-Aware Cooperation Model

In the considered sensor network, the cooperative security node may not always provide the same level of jamming assistance. Its response can depend on the available energy budget, the cooperation relationship with legitimate nodes, and the attack-risk indication provided by a trust-estimation or attack-detection interface. Following the trust-aware relay attack model in Section 2.2, the coefficient τ∈[0,1] is used to represent the security-response level of node *J*. A larger τ indicates a stronger willingness or capability of the cooperative security node to participate in cooperative jamming.

In practical sensor-network deployments, τ can be determined according to the trust score of the relay sensor, historical forwarding behavior, physical-layer observations, or the detection output provided by an external attack-detection interface. In this work, we focus on the physical-layer security response under a given τ, while the detailed design of the trust-estimation mechanism is left as a possible extension.

To incorporate the trust-aware cooperation behavior into the jamming model, we introduce a non-decreasing cooperation function ω(τ), satisfying

(14) 0≤ω(τ)≤1.

Unless otherwise specified, the following linear model is adopted

(15) ω(τ)=τ.

The effective artificial-noise power radiated through the selected pinching antennas is then defined as

(16) $$Q_{J}≜P_{J}^{\mathrm{eff}}=\omega \left(\tau \right)P_{J},$$

where $P_{J}$ denotes the nominal jamming-power variable before trust-aware scaling, and $P_{J}^{\max}$ denotes its maximum available value. The variable $Q_{J}$ represents the actual effective jamming power used in the physical-layer security response. This model includes the non-cooperative case ω(τ)=0 and the fully cooperative case ω(τ)=1 as two special cases.

### 3.2. Equivalent Transmission Rates for Secure Sensor Transmission

Based on the first-phase signal model, the signal-to-interference-plus-noise ratio (SINR) at the untrusted relay sensor is given by

(17) $$\gamma_{R}=\frac{P_{S}{\left|h_{S,R}\right|}^{2}}{Q_{J}{\left|g_{R}\left(a\right)\right|}^{2}+\sigma_{R}^{2}}.$$

Since the relay sensor may be compromised or honest-but-curious, its first-phase received signal is treated as a potential source of information leakage. The corresponding upper-bound leakage rate is defined as

(18) $$R_{R}=\frac{1}{2}\log_{2}\left(1+\gamma_{R}\right),$$

where the factor 1/2 accounts for the two-phase half-duplex transmission.

The first-phase SINR of the direct sensing-data link at the destination fusion center is given by

(19) $$\gamma_{D}^{\left(1\right)}=\frac{P_{S}{\left|h_{S,D}\right|}^{2}}{Q_{J}{\left|g_{D}\left(a\right)\right|}^{2}+\sigma_{D}^{2}}.$$

This expression captures the fact that the artificial noise radiated through the selected pinching antennas may also leak to the legitimate fusion center.

In the second phase, the relay sensor forwards a re-encoded signal to the destination fusion center. The corresponding relay-to-destination SINR is modeled as

(20) $$\gamma_{D}^{\left(2\right)}=\frac{\zeta P_{R}{\left|h_{R,D}\right|}^{2}}{\sigma_{D}^{2}},$$

where 0≤ζ≤1 is an equivalent forwarding-quality factor that summarizes the useful information preserved by the lossy or partial forwarding operation at the relay sensor. When ζ=1, the forwarded signal is ideal, whereas a smaller ζ represents stronger forwarding distortion, partial forwarding loss, or imperfect re-encoding. This abstraction is used for comparative evaluation of PASS-assisted jamming strategies and is not intended to provide a closed-form information-theoretic capacity expression for a specific decode-and-forward, amplify-and-forward, or compress-and-forward relay implementation.

For tractability, we adopt an equivalent two-branch combining model at the destination fusion center. The first branch corresponds to the direct observation in Phase 1, while the second branch corresponds to the useful information preserved in the re-encoded relay signal in Phase 2. Under independent receiver noises and equivalent soft combining, the combined useful signal-to-noise-plus-interference ratio is approximated by the sum of the two branch SINRs. Therefore, the equivalent legitimate sensing-data rate is modeled as

(21) $$R_{D}=\frac{1}{2}\log_{2}\left(1+\gamma_{D}^{\left(1\right)}+\gamma_{D}^{\left(2\right)}\right).$$

The instantaneous secrecy rate is then defined as

(22) $$R_{s}={\left[R_{D}-R_{R}\right]}^{+},$$

where ${\left[x\right]}^{+}=\max\left(x,0\right)$.

### 3.3. Reliable-and-Secure Probability and Secrecy Outage

In security-critical sensor networks, successful transmission requires both reliable data delivery and confidentiality protection. Therefore, we adopt the RSP as the main performance metric. It is defined as

(23) $$P_{\mathrm{RSP}}=\mathrm{Pr}\left(R_{D}\geq R_{\mathrm{th}},R_{s}\geq R_{s}^{\mathrm{th}}\right),$$

where $R_{\mathrm{th}}$ is the target reliable sensing-data transmission rate and $R_{s}^{\mathrm{th}}$ is the target secrecy rate.

The SOP is defined as

(24) $$P_{\mathrm{SOP}}=\mathrm{Pr}\left(R_{s}<R_{s}^{\mathrm{th}}\right).$$

While $P_{\mathrm{RSP}}$ jointly evaluates reliability and secrecy, $P_{\mathrm{SOP}}$ focuses on the confidentiality loss caused by information leakage at the untrusted relay sensor.

For a given PASS activation vector a and effective jamming power $Q_{J}$, both $P_{\mathrm{RSP}}$ and $P_{\mathrm{SOP}}$ are evaluated over channel fading realizations and, when applicable, random sensor-node locations. These metrics are used to quantify how the trust-aware PASS-assisted security response improves secure sensing-data transmission under relay attack risks.

### 3.4. Attack-Aware Secure Delivery Probability

To explicitly evaluate the effect of relay attacks and detection-triggered security response, we define an evaluation metric termed the attack-aware secure delivery probability. Based on the relay security state $\theta_{R}$ defined in Section 2.2, let

(25) $$p_{a}=\mathrm{Pr}\left(\theta_{R}=1\right)$$

denote the probability that the relay sensor is compromised or suspicious. When $\theta_{R}=0$, successful delivery requires reliable sensing-data transmission. When $\theta_{R}=1$, successful delivery requires both reliability and secrecy.

For a given PASS activation vector a and effective jamming power $Q_{J}$, the reliability-only probability is defined as

(26) $$P_{\mathrm{rel}}\left(a,Q_{J}\right)=\mathrm{Pr}\left(R_{D}\geq R_{\mathrm{th}}\right).$$

Let $P_{\det}$ and $P_{\mathrm{fa}}$ denote the attack detection probability and false alarm probability of an abstract attack-detection interface, respectively. The proposed attack-aware PASS scheme switches between a low-response mode and a high-response mode according to the detection output. The corresponding attack-aware secure delivery probability, denoted by $P_{\mathrm{SDP}}$, is expressed as

(27) $$\begin{array}{c} P_{\mathrm{SDP}}=\left(1-p_{a}\right)\left[\left(1-P_{\mathrm{fa}}\right)P_{\mathrm{rel}}^{\mathrm{low}}+P_{\mathrm{fa}}P_{\mathrm{rel}}^{\mathrm{high}}\right]+p_{a}\left[\left(1-P_{\det}\right)P_{\mathrm{RSP}}^{\mathrm{low}}+P_{\det}P_{\mathrm{RSP}}^{\mathrm{high}}\right]. \end{array}$$

Here, the superscripts “low” and “high” represent the low-response and high-response PASS-assisted security modes, respectively. The false-alarm term $P_{\mathrm{fa}}P_{\mathrm{rel}}^{\mathrm{high}}$ represents the case where the relay is normal but the high-response mode is unnecessarily activated. In this case, stronger artificial-noise radiation may reduce the reliability of the destination fusion center because of additional interference leakage. Conversely, the missed-detection term $\left(1-P_{\det}\right)P_{\mathrm{RSP}}^{\mathrm{low}}$ represents the case where the relay is compromised but only the low-response mode is used. Specifically, the low-response mode uses $\tau_{\mathrm{low}}$ and the corresponding optimized pair $\left(a^{\mathrm{low}},Q_{J}^{\mathrm{low}}\right)$, whereas the high-response mode uses $\tau_{\mathrm{high}}$ and the corresponding optimized pair $\left(a^{\mathrm{high}},Q_{J}^{\mathrm{high}}\right)$. Accordingly, $P_{\mathrm{rel}}^{\mathrm{low}}$, $P_{\mathrm{rel}}^{\mathrm{high}}$, $P_{\mathrm{RSP}}^{\mathrm{low}}$, and $P_{\mathrm{RSP}}^{\mathrm{high}}$ are evaluated under their respective PASS activation and jamming-power settings.

## 4. Problem Formulation and Trust-Aware PASS Security Algorithm

### 4.1. Empirical Security Metrics

Recall that the effective artificial-noise power radiated through the selected pinching antennas of the cooperative security node is given by

(28) $$Q_{J}=P_{J}^{\mathrm{eff}}=\omega \left(\tau \right)P_{J}.$$

Since the nominal jamming power satisfies $0\leq P_{J}\leq P_{J}^{\max}$, the feasible range of $Q_{J}$ is given by

(29) $$0\leq Q_{J}\leq Q_{\max}\left(\tau \right),$$

where

(30) $$Q_{\max}\left(\tau \right)=\omega \left(\tau \right)P_{J}^{\max}.$$

This constraint captures the fact that the actual security response is jointly limited by the jammer power budget and the trust-aware cooperation level.

For a given PASS activation vector a and effective jamming power $Q_{J}$, the reliable-and-secure probability is estimated by Monte Carlo sampling as

(31) $${\hat{P}}_{\mathrm{RSP}}\left(a,Q_{J}\right)=\frac{1}{N_{\mathrm{mc}}}\sum_{n=1}^{N_{\mathrm{mc}}}1\left\{R_{D}^{\left(n\right)}\geq R_{\mathrm{th}},R_{s}^{\left(n\right)}\geq R_{s}^{\mathrm{th}}\right\},$$

where $N_{\mathrm{mc}}$ is the number of channel realizations and 1{·} denotes the indicator function.

Similarly, the secrecy outage probability is estimated as

(32) $${\hat{P}}_{\mathrm{SOP}}\left(a,Q_{J}\right)=\frac{1}{N_{\mathrm{mc}}}\sum_{n=1}^{N_{\mathrm{mc}}}1\left\{R_{s}^{\left(n\right)}<R_{s}^{\mathrm{th}}\right\}.$$

The RSP metric evaluates whether the sensor data transmission is both reliable and secure, whereas the SOP metric focuses on the confidentiality loss caused by information leakage at the untrusted relay sensor.

### 4.2. Optimization Problem

The primary trust-aware PASS security design problem is formulated as the maximization of the empirical reliable-and-secure probability

(33) $$\begin{array}{cc} P_{\mathrm{RSP}}:\;\max_{a,Q_{J}}\; & {\hat{P}}_{\mathrm{RSP}}\left(a,Q_{J}\right)\\ s.t.\; & 1\leq \sum_{m=1}^{M}a_{m}\leq K_{\max},\\ \; & a_{m}\in \left\{0,1\right\},\;\forall m\in M,\\ \; & 0\leq Q_{J}\leq Q_{\max}\left(\tau \right). \end{array}$$

For SOP-oriented evaluation, the same PASS activation and power-allocation framework can be configured to minimize the empirical secrecy outage probability

(34) $$\begin{array}{cc} P_{\mathrm{SOP}}:\;\min_{a,Q_{J}}\; & {\hat{P}}_{\mathrm{SOP}}\left(a,Q_{J}\right)\\ s.t.\; & 1\leq \sum_{m=1}^{M}a_{m}\leq K_{\max},\\ \; & a_{m}\in \left\{0,1\right\},\;\forall m\in M,\\ \; & 0\leq Q_{J}\leq Q_{\max}\left(\tau \right). \end{array}$$

Both problems are mixed-integer stochastic optimization problems. The binary activation vector a determines the PASS-assisted jamming channels toward the untrusted relay sensor and the destination fusion center, while the continuous variable $Q_{J}$ controls the strength of the trust-aware physical-layer security response. Exhaustive search over all possible PASS activation patterns requires

(35) ∑k=1KmaxMk

activation-pattern evaluations, which becomes impractical when the number of candidate pinching-antenna positions is large.

To handle both RSP maximization and SOP minimization in a unified manner, we define the following empirical utility function:

(36) $$\hat{U}\left(a,Q_{J}\right)=\left\{\begin{array}{cc} {\hat{P}}_{\mathrm{RSP}}\left(a,Q_{J}\right),\; & \mathrm{RSP}\text{-}\mathrm{oriented}\;\mathrm{mode},\\ -{\hat{P}}_{\mathrm{SOP}}\left(a,Q_{J}\right),\; & \mathrm{SOP}\text{-}\mathrm{oriented}\;\mathrm{mode}. \end{array}\right.$$

Thus, the proposed algorithm always selects the PASS activation pattern and effective jamming power that maximize $\hat{U}\left(a,Q_{J}\right)$.

### 4.3. Candidate-Set-Aided PASS Activation

To reduce the computational complexity while maintaining a strong security performance, we propose a candidate-set-aided PASS security algorithm. Instead of exhaustively checking all possible activation patterns, the algorithm constructs a compact candidate set containing several representative PASS activation patterns and then selects the best one according to the empirical utility function.

Let $a_{A}$ denote the binary activation vector corresponding to the active set A. For each candidate active set A, the optimal effective jamming power is obtained by one-dimensional grid search

(37) $$Q_{J}^{★}\left(A\right)=\arg\max_{Q_{J}\in Q}\hat{U}\left(a_{A},Q_{J}\right),$$

where

(38) $$Q=\left\{0,\Delta Q,2\Delta Q,\ldots ,Q_{\max}\left(\tau \right)\right\}$$

is the discrete search set for the effective jamming power.

The proposed candidate set consists of the following activation patterns.

#### 4.3.1. Metric-Driven Greedy Candidate

The metric-driven greedy candidate is generated by directly evaluating the empirical utility function. Starting from A=∅, the algorithm repeatedly adds one pinching antenna. At each step, the best antenna is selected as

(39) $$m^{★}=\arg\max_{m\in M\setminus A}\max_{Q_{J}\in Q}\hat{U}\left(a_{A\cup \{m\}},Q_{J}\right).$$

The active set is updated only if the empirical utility improves. This process continues until the utility no longer improves or the activation constraint $\left|A\right|=K_{\max}$ is reached.

#### 4.3.2. Relay-Oriented Candidate

The relay-oriented candidate selects pinching antennas that create strong jamming channels toward the untrusted relay sensor. The relay-oriented score of the *m*-th pinching antenna is defined as

(40) $$s_{m}^{R}={\left|\rho_{m}{\tilde{h}}_{m,R}\right|}^{2}.$$

The antennas with the largest relay-oriented scores are selected. This candidate serves as a trust-unaware baseline because it focuses only on suppressing the relay sensor and does not explicitly account for the interference leakage to the destination fusion center.

#### 4.3.3. Destination-Protection Candidate

To avoid excessive artificial-noise leakage to the destination fusion center, we also construct a destination-protection candidate. For a current active set A, the marginal utility of adding the *m*-th pinching antenna is defined as

(41) $$\begin{array}{c} \Delta_{m}\left(A\right)=\frac{{\left|g_{R}\left(a_{A\cup \{m\}}\right)\right|}^{2}-{\left|g_{R}\left(a_{A}\right)\right|}^{2}}{\sigma_{R}^{2}}-\eta \frac{{\left|g_{D}\left(a_{A\cup \{m\}}\right)\right|}^{2}-{\left|g_{D}\left(a_{A}\right)\right|}^{2}}{\sigma_{D}^{2}}, \end{array}$$

where η≥0 is a weighting factor that controls the protection of the destination from excessive artificial-noise leakage. In the simulations, η is set to $\eta_{0}=1.5$. The pinching antenna with the largest positive marginal utility is added at each step until the activation constraint is reached or no positive marginal utility remains.

#### 4.3.4. Random and Warm-Start Candidates

To improve robustness against local greedy decisions, several random activation patterns satisfying

(42) $$1\leq \left|A\right|\leq K_{\max}$$

are included in the candidate set. When the algorithm is executed repeatedly over different values of τ, $P_{J}^{\max}$, or $K_{\max}$, the previously selected active set can also be included as a warm-start candidate.

Let C denote the final candidate set. The proposed PASS activation and power-allocation solution is obtained as

(43) $$\left(A^{★},Q_{J}^{★}\right)=\arg\max_{A\in C,\;Q_{J}\in Q}\hat{U}\left(a_{A},Q_{J}\right).$$

Algorithm 1 summarizes the complete candidate-generation and selection procedure.
**Algorithm 1** Trust-Aware Candidate-Set-Aided PASS Security Response  1:Initialize the candidate set C=∅.  2:Obtain the trust-aware cooperation coefficient τ from a trust-estimation or attack-risk interface.  3:Compute ω(τ) and $Q_{\max}\left(\tau \right)=\omega \left(\tau \right)P_{J}^{\max}$.  4:Construct the power search grid $Q=\{0,\Delta Q,\ldots ,Q_{\max}\left(\tau \right)\}$.  5:Generate the metric-driven greedy candidate and add it to C.  6:Generate the relay-oriented candidate and add it to C.  7:Generate the destination-protection candidate and add it to C.  8:Generate random activation candidates satisfying $1\leq \left|A\right|\leq K_{\max}$ and add them to C.  9:**if** a previous active set is available **then**10:   Add the previous active set to C if it satisfies the activation constraint.11:**end if**12:Remove duplicated activation patterns from C.13:**for** each candidate active set A∈C **do**14:   Compute $g_{R}\left(a_{A}\right)$ and $g_{D}\left(a_{A}\right)$.15:   Obtain $Q_{J}^{★}\left(A\right)=\arg\max_{Q_{J}\in Q}\hat{U}\left(a_{A},Q_{J}\right)$.16:   Record the corresponding utility value.17:**end for**18:Select $A^{★}$ and $Q_{J}^{★}$ with the maximum utility value.19:Evaluate ${\hat{P}}_{\mathrm{RSP}}$ and ${\hat{P}}_{\mathrm{SOP}}$ using $A^{★}$ and $Q_{J}^{★}$.20:Output $A^{★}$, $a^{★}$, $Q_{J}^{★}$, ${\hat{P}}_{\mathrm{RSP}}$, and ${\hat{P}}_{\mathrm{SOP}}$.

### 4.4. Computational Complexity

The exhaustive search method requires evaluating all possible activation patterns and all power levels in Q, leading to a complexity proportional to

(44) ONmc|Q|∑k=1KmaxMk.

In contrast, the proposed candidate-set-aided method evaluates only a limited number of candidate activation patterns. The metric-driven greedy search requires at most $K_{\max}M$ candidate trials, and the final candidate evaluation requires |C| power searches. Therefore, the overall complexity is approximately

(45) $$O\left(N_{\mathrm{mc}}\left|Q\right|\left(K_{\max}M+\left|C\right|\right)\right),$$

which is significantly lower than exhaustive search when *M* is large and $K_{\max}\ll M$. The proposed candidate-set-aided algorithm is a heuristic method and does not guarantee global optimality. Its purpose is to reduce the activation-pattern search space while preserving several representative candidate structures, including metric-driven, relay-oriented, destination-protection, random, and warm-start patterns. The performance gap to the global optimum is not analytically bounded in this work and is left for future investigation.

The added cooperative security node requires one artificial-noise RF source, the dielectric waveguide, and PASS activation control. Its baseband task is payload-independent artificial-noise generation together with the candidate and power search quantified above; it does not decode or re-encode the sensing payload. A source- or relay-side coding alternative could avoid this jamming hardware, but would instead introduce code-dependent encoding/decoding operations, redundancy, latency, and codebook or key coordination at data-aware nodes. A numerical operation-count comparison would depend on the selected channel or secrecy code and its block length, which are not specified in the present system model. The proposed design therefore trades additional RF/PASS hardware for a cooperative node that does not require access to the confidential payload; only the algorithmic complexity of the proposed PASS control is quantified in this work.

## 5. Numerical Results

In this section, numerical results are presented to evaluate the proposed trust-aware PASS-assisted security response for intelligent sensor networks with a potentially compromised relay sensor. The proposed scheme is compared with the following benchmark schemes:

- Fixed-antenna jamming: the cooperative security node uses a single fixed radiating point instead of PASS-based spatial activation;
- Random PASS activation: the activated pinching antennas are randomly selected under the same activation constraint;
- Trust-unaware PASS: the pinching antennas are selected mainly according to the jamming strength toward the relay sensor, without explicitly considering the trust-aware security response or the interference leakage to the fusion center;
- No jamming: no artificial noise is radiated by the cooperative security node.

For the relay-attack evaluation, we further include a No-detection PASS benchmark, where the cooperative security node uses a fixed security response without detection-triggered mode switching. The trust-unaware PASS benchmark corresponds to the relay-oriented activation rule alone. Since the proposed candidate-set-aided algorithm also includes this relay-oriented pattern as one candidate, the comparison evaluates whether additional destination-protection, metric-driven, random, and warm-start candidates can provide further performance improvement beyond this baseline.

It should also be clarified that the no-jamming scheme is used only as an external reference benchmark. It is not a feasible solution of the PASS-assisted jamming optimization problems in (33) and (34), where at least one pinching antenna is activated when cooperative jamming is enabled. In the no-jamming benchmark, the artificial-noise power is set to zero and no PASS activation is performed.

### 5.1. Simulation Setup

The source sensor *S*, the untrusted relay sensor *R*, the destination fusion center *D*, and the PASS-enabled cooperative security node *J* are deployed in a two-dimensional area. Unless otherwise specified, their coordinates are set as S=(0,0), R=(45,8), and D=(90,0) meters. The PASS waveguide is placed parallel to the horizontal axis with candidate pinching-antenna positions uniformly distributed from (30,18) to (80,18) meters. The direct wireless links experience distance-dependent path loss and Rician small-scale fading, while the PASS-assisted jamming links are modeled as line-of-sight-dominated links.

Unless otherwise specified, the carrier frequency is 60 GHz, the system bandwidth is 10 MHz, the noise figure is 7 dB, and the transmit powers of the source sensor and relay sensor are both 20 dBm. The 60 GHz carrier frequency is used to represent a millimeter-wave sensing-communication scenario where PASS-enabled spatial channel shaping is meaningful. Since the purpose of the simulation is to isolate the impact of PASS activation and cooperative jamming, a narrowband equivalent channel with 10 MHz bandwidth is adopted. Therefore, the results should be interpreted as subband-level comparative performance rather than full wideband link-budget validation. The maximum jamming power is set to $P_{J}^{\max}=5$ dBm, except in Figure 3, where it is varied. The number of candidate pinching-antenna positions is M=24, and the maximum number of activated pinching antennas is $K_{\max}=4$, except in Figure 4, where the impact of $K_{\max}$ is investigated. The target reliable transmission rate and target secrecy rate are set to $R_{\mathrm{th}}=1.8$ bps/Hz and $R_{s}^{\mathrm{th}}=0.7$ bps/Hz, respectively. The results are obtained by Monte Carlo simulation with $N_{\mathrm{mc}}=10^{4}$ channel realizations.

The numerical evaluation was implemented in MATLAB R2025b Update 4 (Version 25.2.0.3150157) using double-precision arithmetic. For each parameter point, the rates in Section 3 and the empirical probabilities in Section 4.1 were evaluated from the stated channel model and $10^{4}$ Monte Carlo realizations. All compared schemes used the same geometry, channel distributions, thresholds, and power constraints. Internal consistency was checked through the model-defined limiting cases: $Q_{J}=0$ reproduces the no-jamming signal model, τ=0 yields $Q_{\max}\left(\tau \right)=0$, and every candidate was checked against $1\leq \left|A\right|\leq K_{\max}$ and $0\leq Q_{J}\leq Q_{\max}\left(\tau \right)$. These checks support the numerical consistency of the simulation data; the results remain model-based predictions rather than hardware measurements. Because no execution-time result is reported, processor-specific timing was not evaluated.

For the attack-aware secure delivery evaluation in Figure 5, the attack detection probability and false alarm probability are set to $P_{\det}=0.9$ and $P_{\mathrm{fa}}=0.08$, respectively. The low-response and high-response cooperation coefficients are set to $\tau_{\mathrm{low}}=0.1$ and $\tau_{\mathrm{high}}=0.8$, while the no-detection PASS benchmark uses a fixed cooperation coefficient $\tau_{\mathrm{fix}}=0.1$. Table 1 summarizes the main simulation parameters used in the numerical evaluation.

### 5.2. Impact of Trust-Aware Cooperation

**Figure 2:**
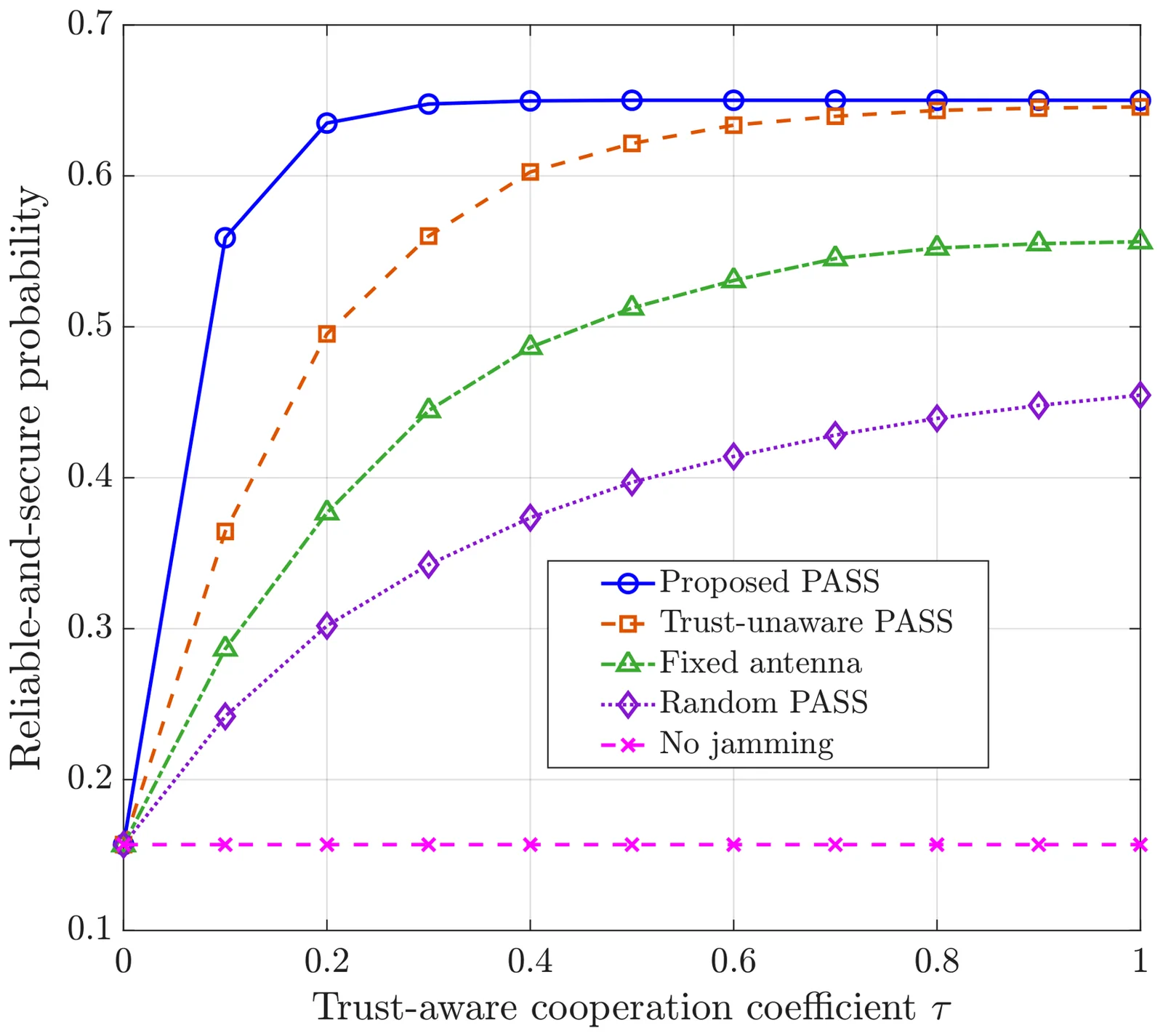
Reliable-and-secure probability versus the trust-aware cooperation coefficient.

Figure 2 shows the reliable-and-secure probability versus the trust-aware cooperation coefficient τ. As τ increases, the effective jamming power budget becomes larger, enabling the cooperative security node to provide a stronger PASS-assisted physical-layer security response against the potentially compromised relay sensor.

The proposed PASS scheme obtains the largest reliable-and-secure probability among the considered schemes over the simulated range of τ. The gain is especially significant in the low-to-moderate cooperation region, where the available jamming capability is limited and careful PASS activation is important. Compared with the trust-unaware PASS benchmark, the proposed scheme improves the secure sensing-data delivery performance by jointly considering the jamming effect on the relay sensor and the interference leakage to the fusion center. When τ becomes large, the performance gradually saturates because the reliable-and-secure probability is jointly limited by the destination reliability, the relay-to-destination forwarding link, and the fixed total jamming power budget.

### 5.3. Impact of Maximum Jamming Power

**Figure 3:**
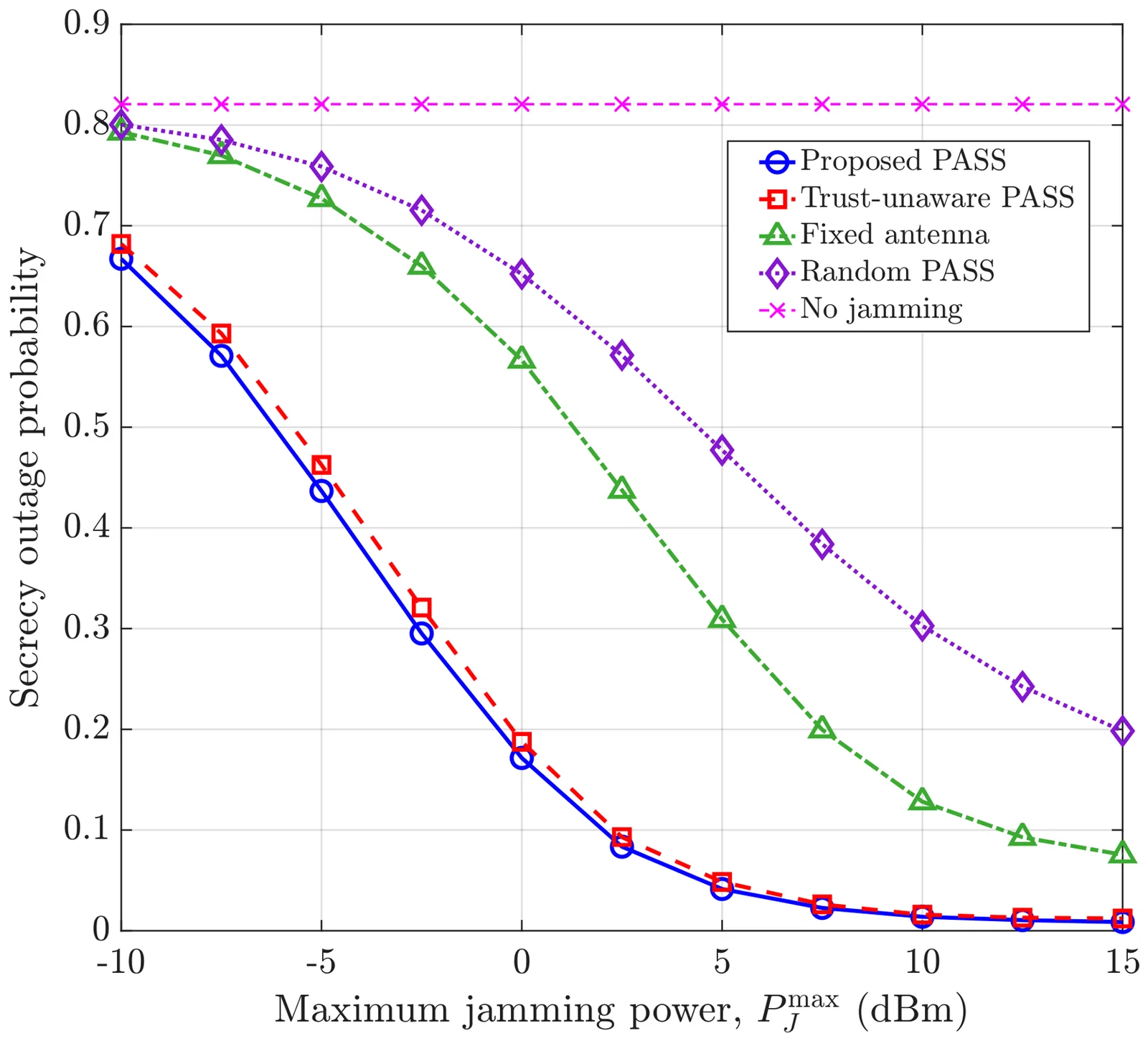
Secrecy outage probability versus the maximum jamming power.

Figure 3 shows the secrecy outage probability versus the maximum jamming power budget $P_{J}^{\max}$ for τ=0.6. As $P_{J}^{\max}$ increases, the secrecy outage probability of the cooperative-jamming schemes decreases because the information leakage at the untrusted relay sensor is more effectively suppressed.

The proposed PASS scheme achieves the lowest secrecy outage probability over the entire power range. This improvement can be attributed to the candidate-set-aided PASS activation, which selects pinching antennas that strengthen the jamming effect toward the relay sensor while limiting artificial-noise leakage to the fusion center. Compared with the trust-unaware PASS benchmark, the improvement is moderate but consistent. The advantage over the fixed-antenna and random-PASS benchmarks is more pronounced, demonstrating the benefit of spatially selective PASS-assisted jamming. The no-jamming scheme remains almost unchanged because it is independent of the jamming power budget.

### 5.4. Impact of the Maximum Number of Activated Pinching Antennas

**Figure 4:**
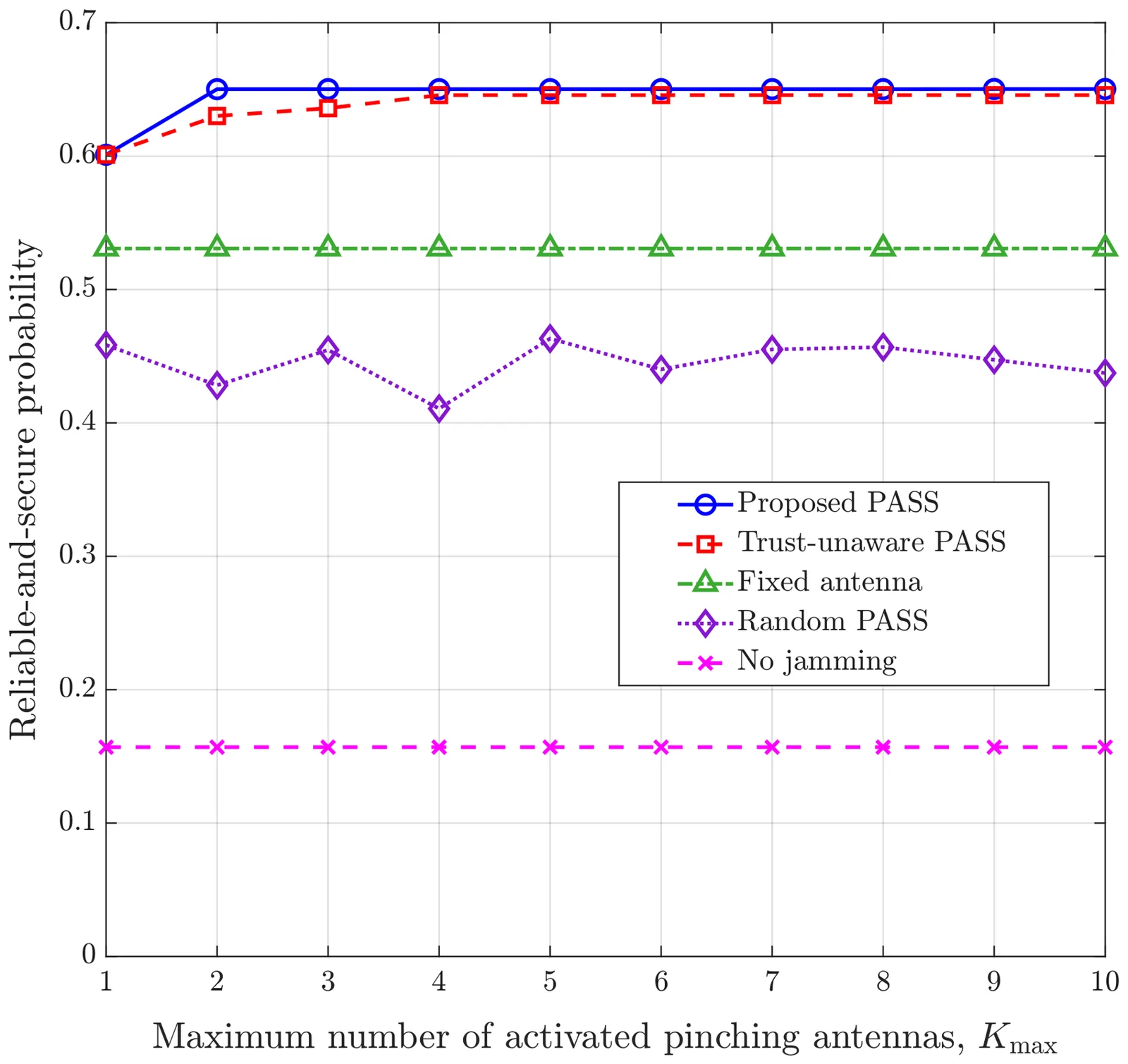
Reliable-and-secure probability versus the maximum number of activated pinching antennas.

Figure 4 shows the reliable-and-secure probability versus the maximum number of activated pinching antennas $K_{\max}$. The proposed PASS scheme achieves a clear gain when $K_{\max}$ increases from 1 to 2, demonstrating the benefit of exploiting spatial flexibility in PASS-assisted jamming.

When $K_{\max}$ further increases, the performance gradually saturates. Under the adopted total-power normalization, activating more pinching antennas does not increase the total radiated artificial-noise power. Therefore, the performance gain mainly comes from additional spatial selection freedom and gradually saturates once a sufficiently effective subset of pinching antennas has been found. Instead, it only provides additional spatial selection freedom. The result indicates that a small number of properly selected pinching antennas can already provide most of the secure transmission gain, which is attractive for low-complexity and low-cost intelligent sensor deployments.

### 5.5. Impact of Relay Attack Probability

**Figure 5:**
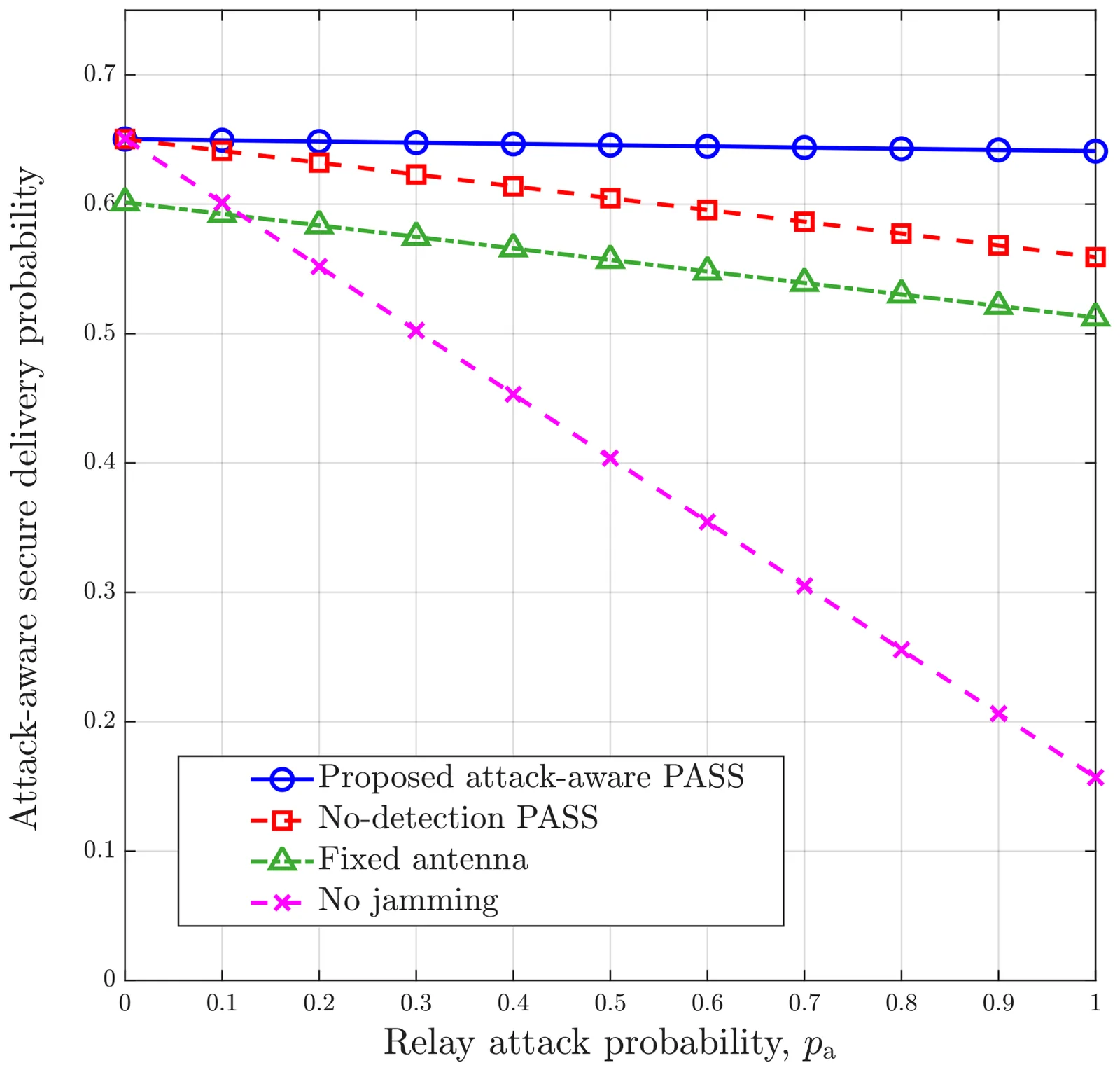
Attack-aware secure delivery probability versus the relay attack probability.

Figure 5 shows the attack-aware secure delivery probability versus the relay attack probability $p_{a}$. In this evaluation, the relay sensor is normal with probability $1-p_{a}$ and compromised with probability $p_{a}$. When the relay sensor is normal, successful delivery requires reliable sensing-data transmission. When the relay sensor is compromised, successful delivery requires both reliability and secrecy. The proposed attack-aware PASS scheme uses an abstract detection output to switch between low-response and high-response security modes. It should be noted that the detection process itself is not optimized in this work; instead, the parameters $P_{\det}$ and $P_{\mathrm{fa}}$ are used to model the reliability of an external or pre-existing attack-detection interface.

Since $P_{\det}$ and $P_{\mathrm{fa}}$ are fixed in this evaluation, Figure 5 should be interpreted as a conditional performance evaluation under a given detection-interface reliability, rather than as a measurement of the detection algorithm itself. The purpose of this result is to quantify how the PASS-assisted physical-layer response benefits secure delivery when a detection output is available.

Under the assumed detection-interface reliability, the proposed attack-aware PASS scheme maintains the largest secure delivery probability among the considered schemes as $p_{a}$ increases. This result suggests that a detection-triggered response can help maintain secure delivery by activating a stronger PASS-assisted security mode when the relay sensor is likely to be compromised. In contrast, the no-detection PASS benchmark uses a fixed security response and therefore gradually degrades as the attack probability increases. The fixed-antenna benchmark performs worse because it lacks PASS-based spatial selectivity. The no-jamming scheme decreases most rapidly because it provides no protection against information leakage at the relay sensor.

It is worth noting that the no-jamming scheme can have a relatively high delivery probability when $p_{a}=0$, since only reliability is required in the absence of relay attacks and no artificial-noise interference is introduced. However, its performance sharply deteriorates as $p_{a}$ increases, which highlights the importance of attack-aware secure transmission in sensor networks.

## 6. Limitations and Practical Considerations

This work focuses on the physical-layer security response after a trust or attack-risk indication has been obtained. The detailed design of the trust-estimation or attack-detection algorithm is outside the scope of this paper. Therefore, the attack-aware secure delivery probability should be interpreted as an evaluation metric for a detection-triggered security response rather than as the performance of a complete attack-detection system.

Second, the numerical results are based on Monte Carlo simulations using a geometry-based channel model and a discretized PASS waveguide. Although this setting is useful for evaluating the proposed PASS activation and jamming-power allocation strategy, experimental validation using software-defined radio platforms, prototype PASS hardware, or realistic sensor-network testbeds is still required before practical deployment. The forwarding-quality factor ζ is fixed in the present numerical evaluation. Since ζ affects the useful information preserved by the relay forwarding operation, additional sensitivity analysis over different ζ values would further clarify the robustness of the proposed scheme.

Third, the present model considers a single relay sensor, a single destination fusion center, and a single cooperative security node. Extensions to multi-relay, multi-sensor, mobile, and multi-attacker scenarios may introduce additional scheduling, coordination, and scalability issues.

Finally, the PASS-assisted jamming links are modeled as line-of-sight-dominated links in the numerical evaluation. More general channel conditions, including blockage, hardware impairments, imperfect channel-state information, and time-varying waveguide losses, should be investigated in future work. In addition, the adopted PASS normalization model treats the selected pinching antennas through an equivalent total-power normalization. This model neglects mutual coupling, non-ideal power extraction, and waveguide loading effects among multiple activated pinching antennas. Therefore, the results should be interpreted as an equivalent baseband evaluation of PASS-assisted jamming rather than a full electromagnetic characterization of practical PASS hardware.

Accordingly, the PASS adopted in this work is an idealized equivalent baseband model: the candidate pinching antennas have prescribed positions and efficiencies, while mutual coupling, impedance mismatch, element-pattern variation, and activation-dependent waveguide loading are not modeled. A different antenna form would change $\eta_{m}$, the element radiation pattern represented in ${\tilde{h}}_{m,u}$, the candidate geometry, and possibly the waveguide attenuation and phase response. These changes would alter both the relay-directed jamming channel and the interference leakage to the fusion center, and could therefore change the selected active set and the resulting RSP and SOP. Quantitative comparison among antenna forms requires hardware-specific electromagnetic parameters and is outside the scope of the present baseband study.

## 7. Conclusions

This paper proposed a trust-aware PASS-assisted secure transmission framework for intelligent sensor networks with a potentially compromised relay sensor. The relay sensor assists sensing-data forwarding but is also treated as a potential source of internal information leakage. To mitigate this threat, the proposed framework uses a PASS-enabled cooperative security node to generate artificial noise, feed it into the PASS waveguide, and radiate it through selected pinching antennas toward the untrusted relay while limiting interference leakage to the destination fusion center.

A trust-aware cooperation coefficient was introduced to characterize the security-response capability of the cooperative security node, and the corresponding effective jamming power was incorporated into the physical-layer transmission model. Reliable-and-secure probability and secrecy outage probability were adopted to jointly evaluate data reliability and confidentiality. A candidate-set-aided PASS activation and jamming-power allocation algorithm was developed for both RSP-oriented and SOP-oriented secure transmission.

Numerical results showed that the proposed scheme improves the reliable-and-secure probability and reduces the secrecy outage probability compared with fixed-antenna jamming, random PASS activation, trust-unaware PASS, and no-jamming benchmarks. The results also demonstrated that only a small number of properly selected pinching antennas can provide most of the secure transmission gain. In addition, the attack-aware evaluation showed that a detection-triggered response enables the proposed PASS-assisted scheme to maintain a higher secure delivery probability as the relay attack probability increases.

Future work will extend the proposed framework to multi-relay and multi-sensor networks, mobility-aware PASS deployment, online trust and attack-risk estimation, and learning-assisted real-time PASS activation. Experimental validation using software-defined radio or prototype PASS hardware is also an important direction for practical intelligent sensor security applications.

## Figures and Tables

**Table 1:** Main simulation parameters.

Parameter	Value
Carrier frequency	60 GHz
Bandwidth	10 MHz
Noise figure	7 dB
Source transmit power $P_{S}$	20 dBm
Relay transmit power $P_{R}$	20 dBm
Maximum jamming power $P_{J}^{\max}$	5 dBm unless otherwise specified
Number of candidate pinching antennas *M*	24
Maximum activated pinching antennas $K_{\max}$	4 unless otherwise specified
Target reliable rate $R_{\mathrm{th}}$	1.8 bps/Hz
Target secrecy rate $R_{s}^{\mathrm{th}}$	0.7 bps/Hz
Forwarding-quality factor ζ	0.7
Number of Monte Carlo realizations $N_{\mathrm{mc}}$	$10^{4}$
Attack detection probability $P_{\det}$	0.9
False alarm probability $P_{\mathrm{fa}}$	0.08
Low-response coefficient $\tau_{\mathrm{low}}$	0.1
High-response coefficient $\tau_{\mathrm{high}}$	0.8
Path-loss exponent	α=2.2
Reference channel gain	$\beta_{0}={\left(\lambda /\left(4\pi d_{0}\right)\right)}^{2}$, $d_{0}=1$ m
Rician factor for direct links	$K_{R}=3$ dB
Waveguide attenuation coefficient	κ=0.015 Np/m
Radiation efficiency	$\eta_{m}=0.8$, ∀m
Guided wavelength	$\lambda_{g}=\lambda /n_{\mathrm{eff}}$
Effective refractive index	$n_{\mathrm{eff}}=1.4$
Power-search grid size	|Q|=61
Destination-protection weight	$\eta_{0}=1.5$
Number of random candidates	20

## Data Availability

The simulation data are generated from the channel model and parameter settings described in this paper.
